# Syphilitic Joint Disease

**Published:** 1901-03-09

**Authors:** 


### SYPHILITIC JOINT DISEASE.

In a very interesting clinical lecture upon Tuberculous Disease of the Knee-joint Mr. Marmaduke Sheild[1] points out the comparative frequency with which joint disease has a syphilitic origin, and incidentally thus explains the sometimes surprising efficacy of Scott's dressing. In coming to a diagnosis between tuberculous and syphilitic disease of the joint, there are, he says, only two distinctive features of the latter most deceptive malady. In the first place, in a syphilitic joint one may be able to feel gummatous masses in the synovial membrane. They may be felt quite as large as nuts in some instances. The second point is that in syphilis the periosteum of the ends of the bones is generally inflamed and tender. If one gets a case of chronic joint disease, associated with tenderness on pressure near the bone ends and nocturnal pains, one must beware of the probability of its being syphilitic. Of course in a child the signs of congenital syphilis are usually easy enough to recognise. There is the depressed nose, the keratitis of the cornea, the notched teeth, the deafness due to nerve lesions. If such a patient as this gets a chronically diseased joint the administration of iodide of potassium will work wonders; and with this remedy is usually combined mercurial applications, with strapping and bandaging. "And now," says Mr. Shield, "I am going to make, what may seem to you, a very curious statement. I told you that chronic tuberculous disease of the knee was not very common in private practice, but chronic syphilitic disease of the knee is not by any means so rare as you may, *à priori*, think. I have seen a considerable number of such cases. They may occur in married ladies in whom the signs of disease are absent or very slight. A woman may marry a syphilitic man and get a slight infection which may pass without notice. There may be some slight sore throat and a rash in the skin which has never been interpreted or detected. The whole matter passes away and nothing more is heard or thought of it until many years have passed; and then this lady will present herself with chronic disease of her knee, and that, being treated by the well-known Scott's dressing and iodides will get entirely well. And do not think that congenital syphilitic disease of the knee is always marked in children by gross signs—I mean notched teeth, badly-developed nose, and so on—because many people with congenital syphilis have really exceedingly slight symptoms of the malady. But there is one test above all others which is important, the use of the ophthalmoscope. Most cases of congenital syphilis have changes in the choroid." If, then, one gets a chronic case of knee disease in people of good family it is at least as likely to be due to the syphilitic virus as to tuberculous disease. A great success in practice may sometimes be made by the prudent and quiet recognition of this fact, and one may sometimes be guided to such a success by the hint given by the ophthalmoscopic signs. The treatment for a case of joint disease supposed to be syphilitic is best accomplished by applying Scott's dressing and giving iodide of potassium internally. One can hardly doubt that the long-reputed efficacy of Scott's dressing in chronic disease of the joints is largely due to the mercury which it contains. At any rate it often proves to be a most effectual remedy, and this may well be due to the admixture, among the ordinary run of joint diseases, of a not inconsiderable proportion of cases which have their origin in the syphilitic virus.

[1] Clinical Journal, Feb. 6.

